# Impact of the First Wave of the COVID-19 Pandemic on New Applications for Long-term Care Insurance in a Metropolitan Area of Japan

**DOI:** 10.2188/jea.JE20210047

**Published:** 2021-06-05

**Authors:** Satoshi Seino, Yu Nofuji, Yuri Yokoyama, Yui Tomine, Mariko Nishi, Toshiki Hata, Shoji Shinkai, Yoshinori Fujiwara, Akihiko Kitamura

**Affiliations:** 1 Research Team for Social Participation and Community Health, Tokyo Metropolitan Institute of Gerontology, Tokyo, Japan; 2 Department of Nutrition Sciences, Kagawa Nutrition University, Saitama, Japan

In epidemiological studies of older Japanese adults, new certifications for long-term care insurance (LTCI)^1^^,^^2^ have often been used as an outcome of functional disability. However, it is unclear how the coronavirus disease (COVID-19) pandemic has affected this outcome and whether it can be reasonably used as an outcome for ongoing cohort studies. Understanding this will provide suggestions for handling this outcome in cohort studies and also indicate support measures for older adults. Thus, we report the impact of the first wave of the COVID-19 pandemic on new applications for LTCI in our metropolitan cohort.

We investigated the applications and certifications for LTCI and mortality information of 15,500 stratified and randomly sampled residents aged 65–84 years of Ota City, Tokyo, in 2016 to verify the effectiveness of community-wide intervention.^3^ Using these data, we calculated the number of new applications for LTCI per 10,000 people every month from January 2018 to July 2020. The World Health Organization (WHO) declared COVID-19 a global pandemic on March 11, 2020. In Japan, the national government declared a nationwide state of emergency from April 7 to May 25, 2020. Thus, we compared the number of new LTCI applications in the period up to February 2020 with the period after March 2020. We applied regression analysis to the number of new LTCI applications from January 2018 to February 2020 and predicted the number of new LTCI applications after March 2020 using the regression equation. Finally, we compared the predicted and actual numbers of new LTCI applications.

**Figure 1.**
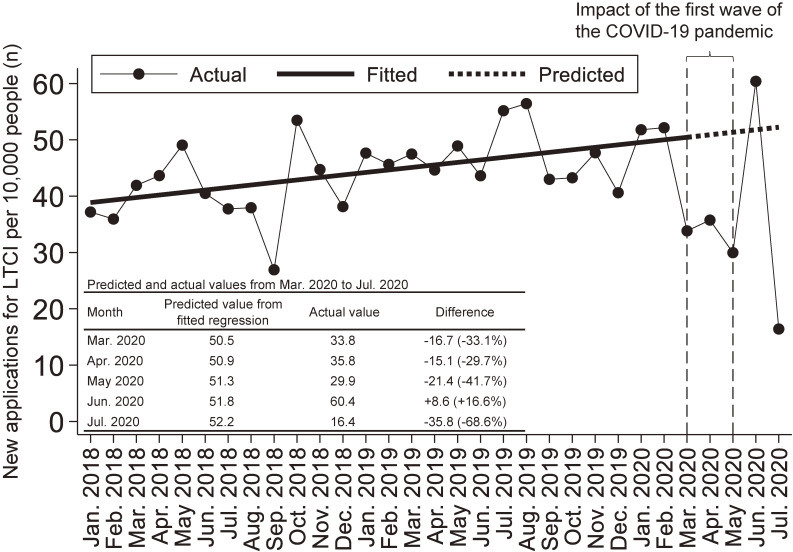
Number of new applications for LTCI per 10,000 people from January 2018 to July 2020 in Ota City cohort. The solid line shows the regression line applied to the number of new LTCI applications from January 2018 to February 2020. The dotted line shows the predicted values based on the regression. The movements of the WHO and the Japanese government regarding COVID-19 are as follows. On January 30, 2020, the WHO declared a public health emergency of international concern. On February 1, 2020, the Japanese government designated COVID-19 as an infectious disease. On March 11, 2020, the WHO declared COVID-19 a global pandemic. On April 7, 2020, the Japanese government declared a nationwide emergency. On May 25, 2020, the Japanese government lifted a nationwide state of emergency. COVID-19, coronavirus disease 2019; LTCI, long-term care insurance; WHO, World Health Organization.

Figure 1 shows the number of new applications for LTCI per 10,000 people monthly from January 2018 to July 2020. According to the parameter estimates from the regression analysis, the number of new LTCI applications gradually increased (by 0.4/10,000 people/month; 95% confidence interval, 0.2–0.7) prior to March 2020. However, it drastically decreased by 29.7–41.7% compared to the predicted values from March to May 2020. Impressively, the number of applications reached its maximum (60.4/10,000 persons, +16.6% compared with the predicted value) in June 2020, after the state of emergency was lifted, and its minimum in July 2020 (16.4/10,000 persons, −68.6% compared with the predicted value), when the second wave of the COVID-19 pandemic began.

These results indicate a clear trade-off between the number of new LTCI applications and the waves of the COVID-19 pandemic. In the valley of the first and second waves of the pandemic (June 2020), the number of applications jumped more than the previous trend. The new LTCI application may have been simply postponed until the nationwide state of emergency was lifted. However, it should be noted that the number of outcomes regarding LTCI may be underestimated during the waves of the COVID-19 pandemic in ongoing cohort studies.

A major explanation for this phenomenon may be that older adults and their families avoided applying for LTCI due to concerns about COVID-19 infections. Future investigations are necessary to clarify the reasons for the decrease in applications. Investigations of regional differences are also required because the COVID-19 pandemic has spread from metropolitan areas to local areas, causing regional differences in infection status, including a time lag. Finally, we should continue to carefully monitor the transition of LTCI applications after the second wave of the pandemic because refraining from various activities due to the COVID-19 pandemic for long periods may lead to functional decline in older adults. As the pandemic continues, it is necessary to discuss how to handle health outcomes regarding LTCI in ongoing cohort studies during the COVID-19 pandemic. Although we cannot generalize conclusions from our results alone, we believe this report can lay the groundwork for further discussion.
