# Erratum for “Impact of the First Wave of the COVID-19 Pandemic on New Applications for Long-term Care Insurance in a Metropolitan Area of Japan”

**DOI:** 10.2188/jea.JE20220061

**Published:** 2022-06-05

**Authors:** Satoshi Seino, Yu Nofuji, Yuri Yokoyama, Yui Tomine, Mariko Nishi, Toshiki Hata, Shoji Shinkai, Yoshinori Fujiwara, Akihiko Kitamura

**Affiliations:** 1 Research Team for Social Participation and Community Health, Tokyo Metropolitan Institute of Gerontology, Tokyo, Japan; 2 Department of Nutrition Sciences, Kagawa Nutrition University, Saitama, Japan

In the original publication of this article,^1^ there was an error in the number of new applications for long-term care insurance (LTCI) per 10,000 people in July 2020. This was due to the new LTCI applications that were not reflected in the LTCI system database at the time of reporting. Table 1 shows the erratum for the main text. Figure 1 shows numbers of new applications for LTCI per 10,000 people from January 2018 to July 2020 in Ota City cohort, with the July 2020 value revised. The authors apologize for these errors.

**Figure 1.**
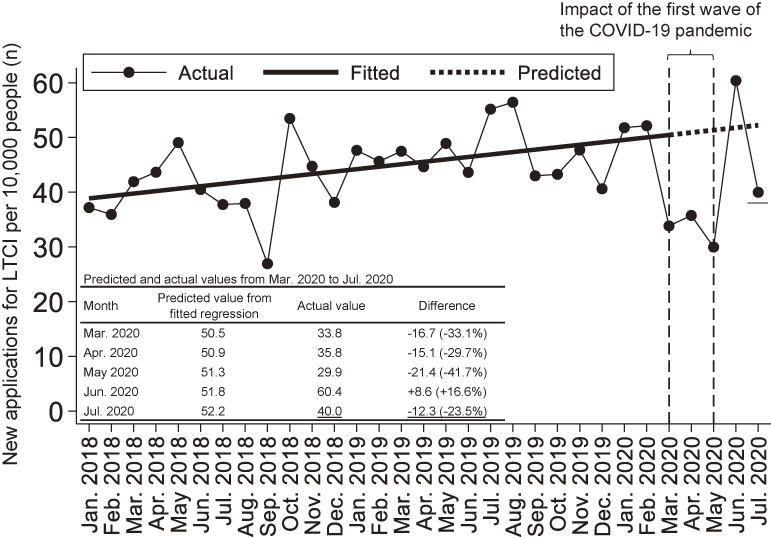
Numbers of new applications for LTCI per 10,000 people from January 2018 to July 2020 in the Ota City cohort, with the July 2020 value revised. The solid line shows the regression line applied to the number of new LTCI applications from January 2018 to February 2020. The dotted line shows the predicted values based on the regression. The actions of WHO and the Japanese government regarding COVID-19 are as follows. On January 30, 2020, the WHO declared a public health emergency of international concern. On February 1, 2020, the Japanese government designated COVID-19 as an infectious disease. On March 11, 2020, the WHO declared COVID-19 a global pandemic. On April 7, 2020, the Japanese government declared a nationwide emergency. On May 25, 2020, the Japanese government lifted a nationwide state of emergency. COVID-19, coronavirus disease 2019; LTCI, long-term care insurance; WHO, World Health Organization.

**Table 1.** Erratum

Before correction	After correction
Main text Impressively, the number of applications reached its maximum (60.4/10,000 persons, +16.6% compared with the predicted value) in June 2020, after the state of emergency was lifted, and **its minimum** in July 2020 (**16.4**/10,000 persons, **−68.6%** compared with the predicted value), when the second wave of the COVID-19 pandemic began.	Main text Impressively, the number of applications reached its maximum (60.4/10,000 persons, +16.6% compared with the predicted value) in June 2020, after the state of emergency was lifted, and **fell** **again** in July 2020 (**40.0**/10,000 persons, **−23.5%** compared with the predicted value), when the second wave of the COVID-19 pandemic began.
